# Direct experimental observation of the gas density depression effect using a two-bunch X-ray FEL beam

**DOI:** 10.1107/S1600577517014278

**Published:** 2018-01-01

**Authors:** Y. Feng, D. W. Schafer, S. Song, Y. Sun, D. Zhu, J. Krzywinski, A. Robert, J. Wu, F.-J. Decker

**Affiliations:** a SLAC National Accelerator Laboratory, 2575 Sand Hill Road, Menlo Park, CA 94025, USA

**Keywords:** gas, density depression, X-ray, FEL, attenuation, pump–probe

## Abstract

The experimental observation of the depression effect in gas devices designed for X-ray free-electron lasers (FELs) is reported. The measurements were carried out at the Linac Coherent Light Source using a two-bunch FEL beam at 6.5 keV with 122.5 ns separation passing through an argon gas cell. The relative intensities of the two pulses of the two-bunch beam were measured, after and before the gas cell, from X-ray scattering off thin targets by using fast diodes with sufficient temporal resolution. At a cell pressure of 140 hPa, it was found that the after-to-before ratio of the intensities of the second pulse was about 17% ± 6% higher than that of the first pulse, revealing lower effective attenuation of the gas cell due to heating by the first pulse and subsequent gas density reduction in the beam path. This measurement is important in guiding the design and/or mitigating the adverse effects in gas devices for high-repetition-rate FELs such as the LCLS-II and the European XFEL or other future high-repetition-rate upgrades to existing FEL facilities.

## Abstract 2

Direct experimental observation of the gas density depression effect by performing an X-ray-pump/X-ray-probe measurement using a two-bunch X-ray FEL beam is presented.

## 1. Introduction   

The phase II project of the Linac Coherent Light Source (LCLS) (Emma *et al.*, 2010 ▸) will seek to upgrade the LCLS conventional accelerator to a superconducting linac, similar to that of the upcoming European XFEL, to generate X-ray free-electron laser (FEL) beams at much higher repetition rates up to 1 MHz. The high-repetition-rate operation of the European XFEL and LCLS-II is expected to provide additional capabilities to those already offered by the low-repetition-rate facilities that are either currently in user operation or being commissioned, including FLASH (Ackermann *et al.*, 2007 ▸) and its upgrade FLASH-II, LCLS, SACLA (Ishikawa *et al.*, 2012 ▸), FERMI (Allaria *et al.*, 2012 ▸), PAL-XFEL (Kang *et al.*, 2013 ▸) and SwissFEL (Ganter *et al.*, 2010 ▸). At the same time, these new capabilities also bring about new challenges in conceiving, designing and implementing high-repetition-rate-compatible X-ray diagnostic, optics, beam regulation and safety devices, which have been proven to be very important to help fulfill FEL’s great scientific potentials in the frontier research of physics, chemistry, life science, material, energy and earth sciences (Young *et al.*, 2010 ▸; Glover *et al.*, 2012 ▸; Fuchs *et al.*, 2015 ▸; Minitti *et al.*, 2015 ▸; Chapman *et al.*, 2011 ▸; Seibert *et al.*, 2011 ▸; Shwartz *et al.*, 2014 ▸; Gerber *et al.*, 2015 ▸; Harmand *et al.*, 2015 ▸; Yoneda *et al.*, 2015 ▸). The ever-so-important X-ray diagnostics for a FEL stems from the stochastic nature of its lasing mechanism, *i.e.* the self-amplified spontaneous emission (SASE) process (Kondratenko & Saldin, 1979 ▸; Bonifacio *et al.*, 1984 ▸), which gives rise to shot-to-shot fluctuations in all beam properties, requiring single-shot diagnostics in pulse energy, timing, spectrum, polarization and more. The successful construction of these diagnostics devices (Richter *et al.*, 2003 ▸; Hau-Riege *et al.*, 2008 ▸; Feng *et al.*, 2011 ▸; Bionta *et al.*, 2011 ▸; Zhu *et al.*, 2012 ▸; Allaria *et al.*, 2014 ▸; Braune *et al.*, 2016 ▸) have allowed users to gain sensitivity in signals which are otherwise difficult to discern, especially those in X-ray pump–probe experiments (Chollet *et al.*, 2015 ▸), as well as enabled FEL operators to tune up the FEL performance and develop new operating modes (Amann *et al.*, 2012 ▸; Marinelli *et al.*, 2013 ▸; Lutman *et al.*, 2014 ▸).

At soft X-ray energies especially, FEL diagnostics and components are often limited to those based on a gas medium to circumvent single-shot damage risks posed by the enormous peak power of a FEL beam. For example, gas intensity monitors and attenuators have been implemented at various facilities including FLASH (Richter *et al.*, 2003 ▸; Hahn & Tiedtke, 2007 ▸), FLASH-II and LCLS (Hau-Riege *et al.*, 2008 ▸; Ryutov *et al.*, 2009 ▸), and are being planned for LCLS-II. The LCLS has the highest operating frequency of all low-repetition-rate facilities, in user operation now or otherwise, at 120 Hz, leaving a time Δ*T* of ∼8.3 ms for the gas system to return to its starting density after the passage of each pulse. A fast energy dissipation has always been implicitly assumed for longer Δ*T*, but this assumption will start to break down as Δ*T* will be reduced by about four orders of magnitude to 222 ns for the European XFEL and 1 µs for LCLS-II. The impact of the very short Δ*T* on the performance of these gas diagnostics and devices has been studied extensively using thermodynamic and hydro­dynamic simulations (Feng *et al.*, 2015*a* ▸,*b* ▸, 2016 ▸; Yang *et al.*, 2017 ▸; Feng & Raubenheimer, 2017 ▸), revealing nonlinear effects in the precision of intensity measurements for gas monitors operating at relatively high pressures (Hau-Riege *et al*., 2008 ▸) as well as the effective attenuation for gas attenuators for trailing pulses, stemming from the density depression or depression phenomenon induced by the energy deposition of the preceding pulses. Parallel efforts have been put forth to experimentally confirm and quantify the depression effect, first by using an optical pump and optical probe technique similar to that used in a previous experiment (Cheng *et al.*, 2013 ▸), and then an X-ray pump and optical probe measurement (Galtier & Schafer, 2017 ▸). It should be noted that for gas monitors operating at extremely low pressures (Richter *et al.*, 2003 ▸) the amount of energy deposited into the medium is very low, and more importantly the absorbed energy is dissipated *via* the detection mechanisms themselves, *i.e.* photoelectrons at the anode and ions neutralized at the cathode in the form of currents.

In this report, we present the first direct experimental observation of the gas depression effect by an ultrafast X-ray FEL beam using an X-ray pump and X-ray probe technique. The measurements were carried out at the LCLS on the X-ray Correlation Spectroscopy (XCS) instrument (Alonso-Mori *et al.*, 2015 ▸) at an X-ray energy of 6.5 keV. Using a special operating mode of the LCLS, a two-bunch FEL beam having two similar intensity femtosecond pulses, which were spatially collinear but separated by over 100 ns in time, was generated. It was sent through the argon-filled gas cell, and the intensities of the two pulses were measured independently, before and after the gas cell. It was found that the transmission of the second pulse was consistently higher than that of the first pulse, revealing a lower effective attenuation consistent with the gas depression effect predicted by thermodynamic and hydro­dynamic calculations (Feng *et al.*, 2015*a* ▸,*b* ▸, 2016 ▸; Yang *et al.*, 2017 ▸; Feng & Raubenheimer, 2017 ▸).

## 2. Experimental setup   

To perform a direct X-ray pump and X-ray probe of the gas depression phenomenon at LCLS before the arrival of LCLS-II, or the availability of a hard X-ray split-and-delay device, the two X-ray pulses were generated using an accelerator-based approach. In a specially developed operating procedure, or the so-called ‘two-bunch operating mode’, two spatially collinear and similar intensity pulses *p*_1_ and *p*_2_ could be produced with *p*_2_ delayed by time Δ*t* amounting to multiples of 0.35 ns (Decker *et al.*, 2010 ▸). For reasons that will become clear later, an optimal delay of order 1 µs would have been ideal for the current experiment, but rather a delay of only 122.5 ns was used due to a limitation of the two-bunch mode. The average intensity of the first pulse *p*_1_ was estimated to be about 1 mJ entering into the gas cell and was purposely tuned to be greater than that of *p*_2_ to maximize the pump and yet to retain sufficient signal-to-noise ratio for the probe.

**Figure 1:**
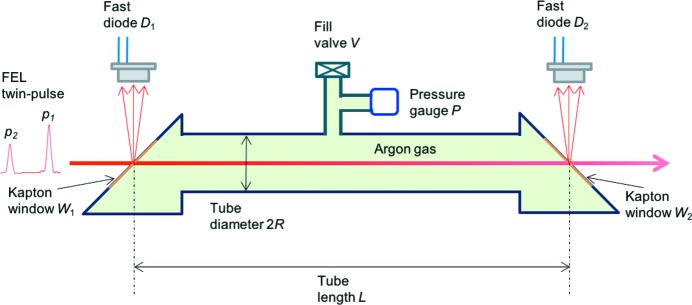
Schematic of the experimental setup, consisting of a gas cell of diameter 2*R* and effective length *L*, filled with argon gas to a pressure *P*. The cell was capped off by two 50 µm-thick Kapton windows *W*_1_ and *W*_2_ each inclined by 45° to the direction of an X-ray two-bunch FEL beam coming from the left and being attenuated. The X-ray scattering off the Kapton windows was measured as the intensities of *p*_1_ and *p*_2_ by two fast diodes *D*_1_ and *D*_2_ positioned perpendicular to the beam and each connected to a different channel of a fast digitizer.

The two-bunch *p*_1_ and *p*_2_ FEL beam was directed through an argon-filled gas cell depicted schematically in Fig. 1 ▸, and attenuated. The gas cell had a diameter 2*R* = 22.1 mm and was sealed off by two 50 µm-thick Kapton windows *W*_1_ and *W*_2_, each inclined at 45° to the direction of the FEL beam and separated by a nominal distance of *L* = 165 ± 8 mm depending on the exact beam path through the cell. The gas pressure *P* was measured *via* an MKS Baratron gauge (Model No. 722B23TGA2FA), with a reading precision of 0.5%, and was varied from *P* = 0 to *P* = 257 hPa. To minimize gas motions not related to the underlying physics to be studied, the gas cell was valved off, but a small (pinhole) leak in the Kapton window developed towards the end of the experiment, leading to a slow creep-up of the gas pressure on a time scale of many tens of minutes. Care was taken to account for the gas leakage by estimating the true effective pressure of the Ar/air mixture by the transmission of a first pulse of the two-bunch beam.

The total X-ray scattering, including both the coherent Thomson, incoherent Compton and other processes, off the Kapton windows was measured *via* two Hamamatsu diodes (Model No. MSM Photodetector G4176-03), *D*_1_ and *D*_2_, positioned perpendicular to the beam. The sides of the diodes were shielded from scattering, but no thin metal foils were used to block any ambient light. The rise time of the diodes was estimated to be of the order of 1 ns, and each diode was connected directly to a different input channel (*via* a 50 Ω coupling resistor) of a fast digitizer (Aquiris 10-bit High-Speed cPCI digitizer, Model No. U1065A). The sampling time was set to 0.25 ns and there was a total of 4000 sampling channels, generating a 1 µs range of digitization. As such, the contribution in the dark noise of the digitizer by ambient light was completely negligible, although any concurrent optical emission upon the passage of the X-ray pulses within the digitization window cannot be separated from the signal. The scattering intensities of *p*_1_ and *p*_2_ were used as measurements of the pulse intensity *I*_1*i*_ and *I*_2*i*_, where *i* = 1 for upstream measurement and *i* = 2 for downstream measurement.

The nominal photon energy of the two-bunch FEL beam was 6.5 keV, with each bunch (pulse) produced independently, and exhibited typical SASE characteristics, including large fluctuations in intensity. The FWHM beam size was measured to be 800 µm in width and 1 mm in height. The two pulses could be steered independently, and care was taken to ensure their spatial overlap over the length of the gas cell by better than 80% of the FWHM size in either transverse direction using a downstream high-resolution beam imager with a spatial resolution of 8 µm. Note that a complete overlap is in principle not achievable due to the SASE nature of FEL beams, which can produce approximately 10% relative spatial jitter pulse-to-pulse and independent of the absolute beam size. The time delay was fixed at 122.5 ns, the maximum achievable with the LCLS accelerator configuration at the time of the measurement. As such, the rise time of the diodes (∼1 ns) was sufficient to resolve the twin pulses, making the data analysis very straightforward without having to be concerned about temporally overlapping signals.

The two-bunch FEL beam size was about 1 mm FWHM before focusing. The formation of the density depression upon passage of a FEL beam takes place over a time scale set by the speed of the shock wave *v*_shock_ (Yang *et al.*, 2017 ▸) at 200 m s^−1^, which is approximately on the same scale as the speed of sound in an ideal gas (*v*_sound_ ≃ 300 m s^−1^). As such, the edge of the density depression only reaches about 24 µm after 122.5 ns and is much smaller than the half-width of the beam at 500 µm, making it much more difficult to observe using the transmission measurement described next. By focusing the beam down to about 100 µm on average over the length of the cell (the spatial jitter is still 10% or 10 µm), clear evidence of an enhanced transmission of the second pulse as induced by the first pulse was found, in qualitative agreement with simulations.

## 3. Data analysis and results   

### 3.1. Transmissions at zero attenuation   

**Figure 2:**
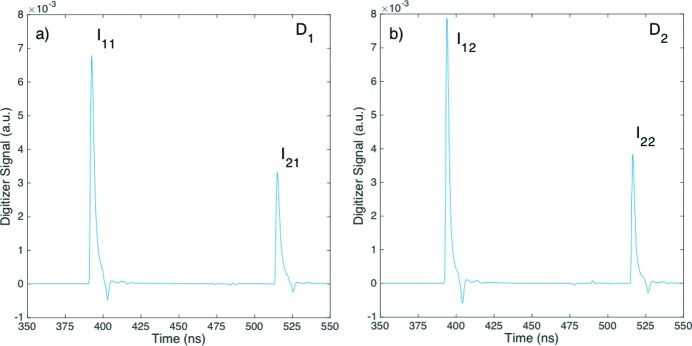
Average digitizer traces of the two-bunch beam measured by (*a*) the upstream diode *D*_1_ and (*b*) the downstream diode *D*_2_ at an argon pressure *P* = 0 hPa. The digitizer was set to sample at 0.25 ns per channel. The integrated intensities of the first pulse *p*_1_ are denoted as *I*_11_ and *I*_12_ on *D*_1_ and *D*_2_, respectively, and those of the second pulse *p*_2_ as *I*_21_ and *I*_22_ on *D*_1_ and *D*_2_, respectively.

The average traces of the pulses *p*_1_ and *p*_2_ from the digitizer of the two-bunch pulses at *P* = 0 hPa are shown in Fig. 2 ▸, with those measured by diode *D*_2_ somewhat larger and at the same time narrower by about 16 ± 1% thus summing up to the same integrated intensity. This is due to the slight difference in the rise time of the two diodes. The average was performed over more than 32000 pulses. There were two contributions to the background, one of random nature and the other periodic and alternating exactly at the first subharmonic of the sampling frequency of 0.25 ns of the digitizer, indicating a digital artifact arising from the clocking scheme. This periodic contribution was simply removed by applying a filter at half of the sampling frequency over the entire sampling range, thus is not visible in Fig. 2 ▸.

The first pulse *p*_1_ and second pulse *p*_2_ were separated by exactly 122.5 ns, and the rise time of the upstream diode *D*_1_ was about 2 ns, whereas for the downstream diode *D*_2_ it was about 1.75 ns, consistent with the 16% difference in their respective amplitudes. The amplitude of the undershoots in traces was also proportional to the main peak, and was caused by a slight impedance mismatch or ringing. The relative intensities of the first pulse *I*_1*i*_ and second pulse *I*_2*i*_ on different diodes *D*_*i*_, for *i* = 1 or 2, were obtained by integrating the digitizer traces over the main peak (not including the undershoot), and the average transmission coefficients of the two pulses *T*_*j*_ = *I*_*j*2_/*I*_*j*1_, for *j* = 1 and 2, were then calculated, obtaining 〈*T*_1_〉 = 1.01 ± 0.02 and 〈*T*_2_〉 = 0.99 ± 0.02, respectively. The near equality of 〈*T*_1_〉 and 〈*T*_2_〉 within the experimental uncertainty was expected as there was no attenuation at *P* = 0 hPa.

**Figure 3:**
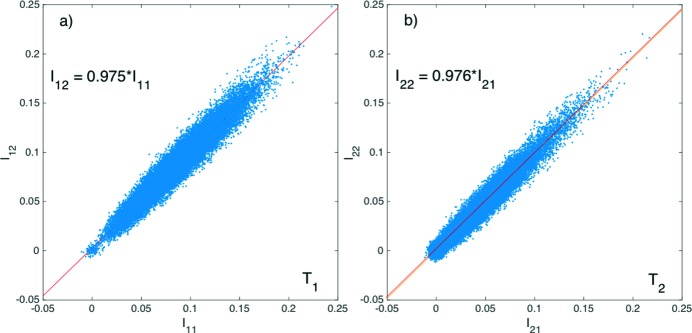
Correlations between the intensities measured by the upstream and downstream diodes of (*a*) the first pulse and (*b*) the second pulse at argon gas pressure *P* = 0 hPa. The slope is a measure of the transmission, amounting to 〈*T*_1_〉 = 97.5% for the first pulse and nearly identical 〈*T*_2_〉 = 97.6% for the second pulse as expected. The very slight difference from unity is considered as a systematic error of the detection scheme. The red line for the first pulse was overlaid in (*b*) to stress the similarities between the transmissions of the two pulses.

The Hamamatsu G4176-03 diode was chosen to achieve sufficient time resolution, but at the expense of having a relatively small dynamic range for detecting hard X-rays. At 6.5 keV, a large number of electron–hole pairs, approximately 1800, are created by a single photon, and the dynamic range was limited to approximately 100 6.5 keV X-rays photons. To avoid saturation, the number of scattered photons into the diodes was purposely limited to less than 100 by adjusting the distances of the diodes to the Kapton windows. As such, large uncertainties were observed in the single-pulse measurement of *I*_*ji*_ for *i*, *j* = 1 and 2, as shown in Figs. 3(*a*) and 3(*b*) ▸ and were attributed to Poisson noise arising from the small number of detected X-ray photons (<100 or a minimum of 10% shot noise). At a given measured relative intensity *I*_11_ or *I*_21_, there is a very wide range of values for *I*_12_ or *I*_22_, with the standard deviation reaching as much as 25%. However, the correlations such as in Fig. 3 ▸ still provided consistent transmission measurements with those obtained from the average traces in Fig. 2 ▸, as given by the slope of a linear fit, at 〈*T*_1_〉 = 0.975 ± 0.003 and 〈*T*_2_〉 = 0.976 ± 0.001 for the first and second pulses, respectively.

### 3.2. Transmissions at finite attenuation   

**Figure 4:**
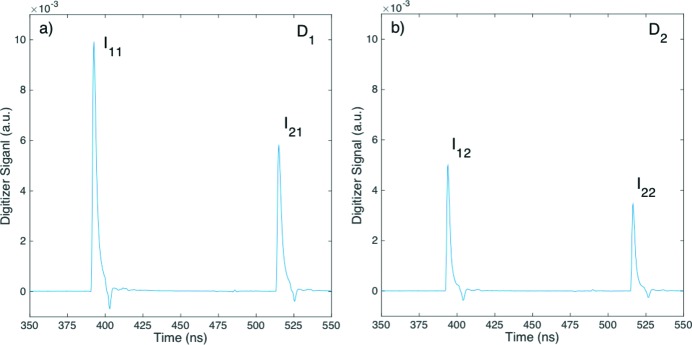
Average digitizer traces of the two-bunch beam measured by (*a*) the upstream diode *D*_1_ and (*b*) the downstream diode *D*_2_ at an effective argon pressure *P* = 140 hPa. The various intensities *I*_*ij*_ are defined previously in Fig. 2 ▸.

Additional measurements similar to those at zero attenuation were repeated at finite attenuations. The average traces of the pulses *p*_1_ and *p*_2_ from the digitizer of the two-bunch pulses at an effective argon pressure of *P* = 140 hPa are shown in Fig. 4 ▸. During the measurement at *P* = 0 hPa, the FEL beam was focused from about 1 mm in size to 100 µm at the location of the gas cell to gain sensitivity. The reading on the pressure gauge at 132 hPa was lower than the actual transmission would indicate; the discrepancy was not completely understood. Again, the systematic periodic background was first removed. Similar to the zero-pressure case, the trace average was performed over more than 35000 pulses. The relative intensities on the downstream diode were lower as expected than those on the upstream but by different amounts in relative terms, indicating different effective transmissions for the two pulses.

**Figure 5:**
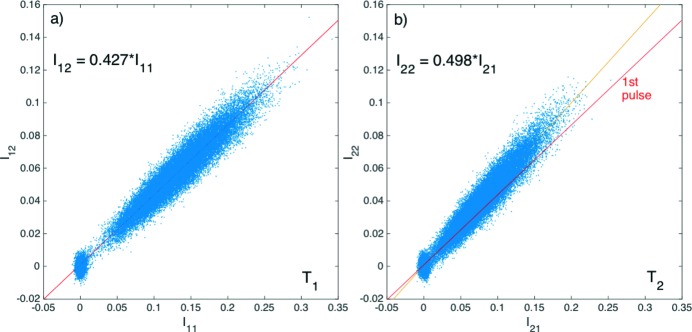
Correlations between the intensities measured by the upstream and downstream diodes of (*a*) the first pulse and (*b*) the second pulse at an equivalent argon gas pressure *P* = 140 hPa. The slope is a measure of the transmission, amounting to 42.7% for the first pulse and an enhanced 49.8% for the second pulse.

**Figure 6:**
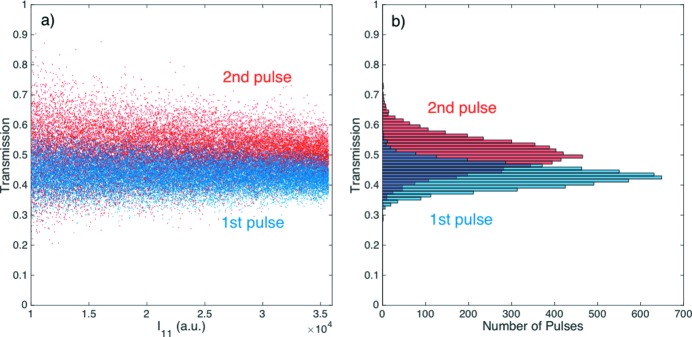
(*a*) Transmission of the first and second pulse sorted based on the intensity of the first or probe pulse *I*_11_, at an effective argon gas pressure *P* = 140 hPa, with an average of 〈*T*_1_〉 = 43% ± 3% and 〈*T*_2_〉 = 50% ± 5% for the first and second pulse, respectively. (*b*) Histogram of the transmission of the first and second pulse. The average transmissions are again 〈*T*_1_〉 = 43% ± 3% and 〈*T*_2_〉 = 50% ± 5% for the first and second pulse, respectively.

The different effective transmissions for the two pulses are best shown by the linear fits of the intensity correlation plots in Fig. 5 ▸, equalling 〈*T*_1_〉 = 0.427 for the first pulse and a higher value 〈*T*_2_〉 = 0.498 for the second pulse. This amounts to a 17% enhancement in the transmission of the second pulse induced by the first pulse. This difference is further highlighted by overlaying the calculated transmissions in Fig. 6(*a*) ▸ sorted by the intensity of the probe pulse *I*_11_, and the transmission histograms in Fig. 6(*b*) ▸, both giving an average of 〈*T*_1_〉 = 43% and 〈*T*_2_〉 = 50% for the first and second pulse, respectively. The large uncertainty in the measurements due to the Poisson noise was again apparent in both figures, with the second pulse having roughly 50% greater noise, consistent with its reduced intensity at 43%, which should scale like 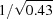 ≃ 1.52.

The enhanced transmission from 〈*T*_1_〉 = 43% to 〈*T*_2_〉 = 50% is much smaller than the 〈*T*_2_〉 = 75% value calculated based on the methodology developed in the previous thermodynamic studies (Feng *et al.*, 2015*a* ▸) and the current experimental geometries. There is a main reason for this discrepancy. The delay time Δ*t* at 122.5 ns was too short so the density depression was not fully developed, or the density depression was not completely formed according to the recent hydro­dynamic studies (Yang *et al.*, 2017 ▸). If using the sound speed of 200 m s^−1^ obtained in the hydro­dynamic simulation and the focused beam size of 100 µm, the largest transmission would occur at approximately 500 ns. As a possible explanation for the smaller than calculated transmission enhancement, one can simply extrapolate linearly the development of the density depression in time, and would arrive at an expected enhanced transmission of 〈*T*_2_〉 = (75% − 43%) × 122.5/500 + 43% = 51%, which is in very good agreement with the measurement. This consistency also supports the time-dependent thermodynamic simulations (Feng *et al.*, 2015*a* ▸ ▸) for time scales beyond the fully opening of the density depression, which depends on the beam size and sound speed. For shorter time scales down to that of the thermalization (Feng *et al.*, 2016 ▸), hydro­dynamic simulations are required to predict the exact transmission. Furthermore, the current experimental measurement also suggests that the main energy transfer mechanism is hydro­dynamic and then thermodynamic, and any radiative losses or other processes may be secondary. More experimental and theoretical studies should be pursued to gain further understanding of the performance of gas devices for high-repetition-rate X-ray FELs.

## 4. Summary   

We have observed experimentally the depression effect in a gas cell induced by an ultrashort X-ray FEL beam using a two-bunch scheme. The observed enhanced transmission of the delayed second pulse in the wake of the first pulse was in good quantitative agreement with the estimate, which was based on thermodynamic simulations and proper extrapolation of the hydro­dynamic simulation results. No enhancement was found as expected when the cell was not filled with an attenuating gas. Our finding is important in guiding the design and/or possibly mitigating the adverse effect in gas devices operating at relatively high pressure for high-repetition-rate FELs such as the LCLS-II, the European XFEL or other future high-repetition-rate upgrades to the existing FEL facilities, and have broader implications not only on gas-based applications but also on liquid- and solid-based devices and experiments.
